# The Moral Basis of Private Property

**Published:** 1890-08-16

**Authors:** 


### The Moral Basis of Private Property.

By the Bishop of London.

### II.—THE GOOD OF THE COMMUNITY.

Now it was unquestionable that it was the accumulation of property which had made material progress possible, and also unquestionable that material progress had stimulated intellectual progress. There would have been no great undertakings in the absence of accumulation. A great harbour was to be constructed, or a great road. These would recoup the expense of construction, but in the meantime, for one or two years, they paid nothing. The labourers must be fed all that time, and this was possible owing to accumulation, and not possible otherwise. To say that all this was immoral was to talk nonsense. To say that such works had arisen from a wrong view and a fundamental mistake in the conduct of the human race, was clearly to ignore what it was that had made the progress of the human race possible at all.

The Socialist might reply—"But have we not gone on long enough with this system based on private property? Can we not find something still better?" Well, what should be the conditions of a community in which there should be no private property at all? Had there been any instance of such a thing? Yes; the thing was common enough, and had done great benefit to mankind. The Monastic orders gave clear evidence of the conditions with which such communities could exist. What were these conditions? Poverty, obedience, and celibacy. These had been found necessary all through their history, and no order had long continued without these three things. So they should examine how far these things were necessary in the community, if a whole country put itself into the position of being without private property. The vow of poverty, for instance, was nothing but socialism put into practice. A man had nothing of his own. The community itself might be rich, but each individual member was to have absolutely nothing. Then, as to the vow of obedience, would anything like this be necessary if England put itself into the position supposed. It would be necessary, and for this reason the community would live by the labour of its members. There were a good many men who did not want to labour; a good many also who liked to choose their own work. The result would be that certain kinds of labour—very necessary, but distasteful—would be altogether uncultivated. Some check would be needed. Then there was the vow of celibacy. Should this be applied to the community? To a certain extent it should. The community possessed a certain amount of goods; and means must be taken to eke out these goods, matters must be regulated according to the mouths which had to be filled. So only a certain number of people could be allowed to get married, and a man must wait his turn, or perhaps not get married at all; to that extent a man would lose the control over his own actions which he possessed at present. These regulations, as applied to the State, also had two disadvantages as compared with their application to the monastic orders; in the latter case everything was voluntarily undertaken, not by compulsion as in the case of the community; and, again, the monastic rule was offered only to people thought fit and suitable for it, whereas in the State the rule must be indiscriminately applied. But supposing, in the case of the State, these vows could be kept, would the keeping of them be for the good of the community? The answer to that was, as history clearly showed, that all the good which had been effected arose from liberty and spontaneous action.

It was sometimes said, as regarded landed property, that God gave the land for the use of everybody, and therefore there should not be an individual ownership in land. Well, were they prepared that a country like China, for instance, with its dense population, should say to themselves—"You must take over so many millions of our people, and provide for them"? That would be carrying out the principle laid down. Again, it was said that if they inquired they often found that land, now in the possession of this or that individual, was wrongly acquired a few generations ago, and therefore should be given up. This would be, at least, to punish the innocent people for the guilty. And there was more to be said about it. Could they go to a labouring man that they found in good employment, with a comfortable home, and say to him: "Your grandfather was my footman, and did not really earn the money he received, and you must therefore cease to enjoy these comfortable surroundings of yours"? Had the community a right to acquire all landed property? The State did at present claim the right to acquire property for the good of the community at large, but, in this case, held to the principle that the community must pay for what was seized. And it would be a strange thing to begin a reign of righteousness by one gigantic act of robbery like a boy at school deciding to begin doing well in earnest by telling an immense lie once for all.

The sweating system also introduced several difficult questions. As regarded the tailor industry, for example, it was quite true that if a man, instead of buying the cheapest garment he could get, gave three times as much for it, that garment might wear five times as long as the cheapest one; and so if the purchaser could save enough for the purpose, he would gain by the dearer garment. But that was not the whole matter. If the workmen got better wages, as things now stood, the master would get no profits, and so this industry would be killed. Was it better, then, to give starvation wages or none at all? Better to give none. A man had a right, if he liked, to buy the cheapest sugar, for example, but not the cheapest labour, because labour was a sacred thing—a man's life, as it were. An employer was certainly not justified in getting the cheapest labour he could.

Two things the Christian minister must teach were—unselfishness to the rich and self-control to the poor. Early marriages were likewise a cause of much poverty and distress. People ought to be influenced morally rather than by compulsion, through their own hearts and consciences rather than by external pressure and forcing.

